# Supplementary material for: A Single-Component Multilayered Self-Assembling Protein Nanoparticle Vaccine Based on Extracellular Domains of Matrix Protein 2 against Both Influenza A and B
Source: Vaccines (Basel). 2024 Aug 28;12(9):975. doi: 10.3390/vaccines12090975 (PMC11435909; doi:10.3390/vaccines12090975)
Supplement: Supplementary file 1 [file vaccines-12-00975-s001.zip › vaccines-3116049-supplementary.pdf]

Figure S1

a **Construct sequence**

>M2exA3B3-5GS-1TD0 (A-A-A-B-B-B)
DAMKRGLCCVLLLCGAVFVSPSQEIHARFRRGARSRSLLTEVETPIRNEWGCRCNDSSDGGGGSLLTEVETPTRNGWESKSSDSSDGGGGSLLTEVETPTRSEWESRSSGSSDGGGGML
EPFQIGGGGMLEPFQIGGGGMLEPFQIGGGGSEVRIFAGNDPAHTATGSSGISSPTPALTPLMLDEATGKLVVWDGQKAGSAVGILVLPLEGTETALTYYKSGTFATEAIHWPESVDEH
KKANAFAGSALSHAA

>M2exA3B3-5GS-I3-01v9a (A-A-A-B-B-B)
DAMKRGLCCVLLLCGAVFVSPSQEIHARFRRGARSRSLLTEVETPIRNEWGCRCNDSSDGGGGSLLTEVETPTRNGWESKSSDSSDGGGGSLLTEVETPTRSEWESRSSGSSDGGGGML
EPFQIGGGGMLEPFQIGGGGMLEPFQIASGGGGSAKLAEELQKKMEELFKKHKIVAVLRANSVEEAKEKALAVFEGGVHLIEITFTVPDADTVIKELSFLKEKGAIIGAGTVTSVEQCR
KAVESGAEFIVSPHLDAEITVFCLEKGVFYMPGVMTPTELVKAMKLGHNILKLFPGEVVGPQFVKAMKGPFPNVKFVPTGGVNLDNVCEWFKAGVLAVGVGSALVKGTPDEVREKAKAF
VEKIRGCTEGGGGSSPAVDIGDRLDELEKALEALSAEDGHDDVGQRLESLLRRWNSRRADGSAKFVAAWTLKAAA

>M2exB3A3-5GS-I3-01v9a (B-B-B-A-A-A)
DAMKRGLCCVLLLCGAVFVSPSQEIHARFRRGARSRMLEPFQIGGGGMLEPFQIGGGGMLEPFQIGGGGSLLTEVETPIRNEWGCRCNDSSDGGGGSLLTEVETPTRNGWESKSSDSSD
GGGGSLLTEVETPTRSEWESRSSGSSDASGGGGSAKLAEELQKKMEELFKKHKIVAVLRANSVEEAKEKALAVFEGGVHLIEITFTVPDADTVIKELSFLKEKGAIIGAGTVTSVEQCR
KAVESGAEFIVSPHLDAEITVFCLEKGVFYMPGVMTPTELVKAMKLGHNILKLFPGEVVGPQFVKAMKGPFPNVKFVPTGGVNLDNVCEWFKAGVLAVGVGSALVKGTPDEVREKAKAF
VEKIRGCTEGGGGSSPAVDIGDRLDELEKALEALSAEDGHDDVGQRLESLLRRWNSRRADGSAKFVAAWTLKAAA

>M2e(AB)x3-5GS-I3-01v9a (A-B-A-B-A-B)
DAMKRGLCCVLLLCGAVFVSPSQEIHARFRRGARSRSLLTEVETPIRNEWGCRCNDSSDGGGGMLEPFQIGGGGSLLTEVETPTRNGWESKSSDSSDGGGGMLEPFQIGGGGSLLTEVE
TPTRSEWESRSSGSSDGGGGMLEPFQIASGGGGSAKLAEELQKKMEELFKKHKIVAVLRANSVEEAKEKALAVFEGGVHLIEITFTVPDADTVIKELSFLKEKGAIIGAGTVTSVEQCR
KAVESGAEFIVSPHLDAEITVFCLEKGVFYMPGVMTPTELVKAMKLGHNILKLFPGEVVGPQFVKAMKGPFPNVKFVPTGGVNLDNVCEWFKAGVLAVGVGSALVKGTPDEVREKAKAF
VEKIRGCTEGGGGSSPAVDIGDRLDELEKALEALSAEDGHDDVGQRLESLLRRWNSRRADGSAKFVAAWTLKAAA

>M2e(BA)x3-5GS-I3-01v9a (B-A-B-A-B-A)
DAMKRGLCCVLLLCGAVFVSPSQEIHARFRRGARSRMLEPFQIGGGGSLLTEVETPIRNEWGCRCNDSSDGGGGMLEPFQIGGGGSLLTEVETPTRNGWESKSSDSSDGGGGMLEPFQI
GGGGSLLTEVETPTRSEWESRSSGSSDASGGGGSAKLAEELQKKMEELFKKHKIVAVLRANSVEEAKEKALAVFEGGVHLIEITFTVPDADTVIKELSFLKEKGAIIGAGTVTSVEQCR
KAVESGAEFIVSPHLDAEITVFCLEKGVFYMPGVMTPTELVKAMKLGHNILKLFPGEVVGPQFVKAMKGPFPNVKFVPTGGVNLDNVCEWFKAGVLAVGVGSALVKGTPDEVREKAKAF
VEKIRGCTEGGGGSSPAVDIGDRLDELEKALEALSAEDGHDDVGQRLESLLRRWNSRRADGSAKFVAAWTLKAAA

b **SDS Page**

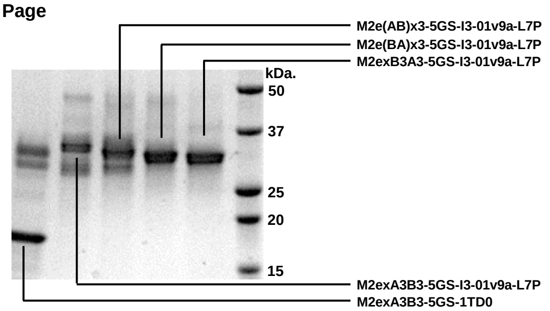

c **nsEM images**

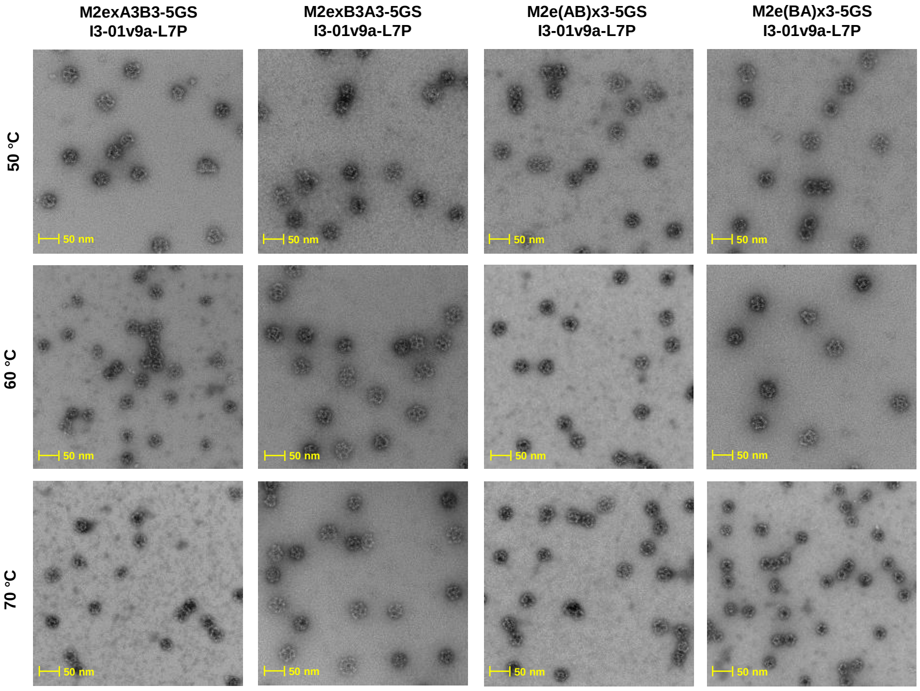

Figure S1

d **ELISA profiles**

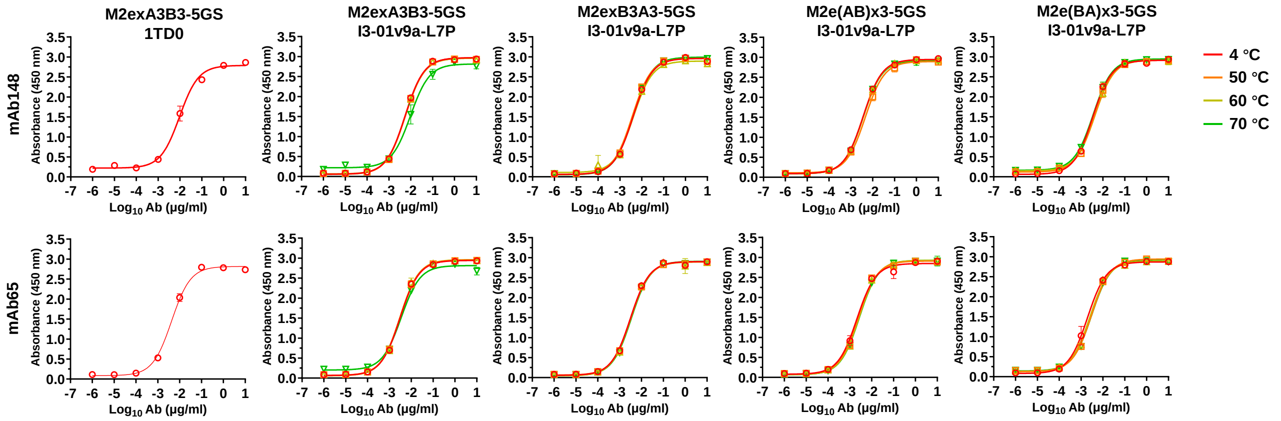

EC$_{50}$ (ug/mL)	mAb148	mAb65
M2exA3B3-5GS-1TD0, 4 °C	0.009586	0.004276
M2exA3B3-5GS-I3-01v9a-L7P, 4 °C	0.00548	0.00306
M2exA3B3-5GS-I3-01v9a-L7P, 50 °C	0.005598	0.003042
M2exA3B3-5GS-I3-01v9a-L7P, 60 °C	0.005624	0.003002
M2exA3B3-5GS-I3-01v9a-L7P, 70 °C	0.00976	0.003431
M2exB3A3-5GS-I3-01v9a-L7P, 4 °C	0.004018	0.003108
M2exB3A3-5GS-I3-01v9a-L7P, 50 °C	0.003661	0.003221
M2exB3A3-5GS-I3-01v9a-L7P, 60 °C	0.004132	0.003153
M2exB3A3-5GS-I3-01v9a-L7P, 70 °C	0.004012	0.003418
M2e(AB)x3-5GS-I3-01v9a-L7P, 4 °C	0.003651	0.002104
M2e(AB)x3-5GS-I3-01v9a-L7P, 50 °C	0.004584	0.002455
M2e(AB)x3-5GS-I3-01v9a-L7P, 60 °C	0.003747	0.002619
M2e(AB)x3-5GS-I3-01v9a-L7P, 70 °C	0.003616	0.002702
M2e(BA)x3-5GS-I3-01v9a-L7P, 4 °C	0.003397	0.001969
M2e(BA)x3-5GS-I3-01v9a-L7P, 50 °C	0.004113	0.002994
M2e(BA)x3-5GS-I3-01v9a-L7P, 60 °C	0.004305	0.002607
M2e(BA)x3-5GS-I3-01v9a-L7P, 70 °C	0.003598	0.003027

Fig. S1. Design and in vitro characterization of influenza A-B M2e-presenting SApNPs. (**a**) Construct sequences of influenza A-B M2e trimer and I3-01v9a-L7P SApNPs, with the gene fragments of sequence, restriction site, flexible linker, human, avian/swine, and human/swine matrix protein 2 extra-virion domain from influenza A virus (residue: 2-24), human matrix protein 2 extra-virion domain from influenza B virus (residue: 2-9), SApNP-forming subunit, trimerization domain (PDB: 1TD0), locking domain (LD), and PADRE highlighted in yellow, teal, green, gray, red, olive green, pink, cyan, and light blue, respectively. (**b**) SDS-PAGE analysis of various influenza A-B M2e trimer and SApNP constructs under reducing conditions. Each well was loaded with 2 μg of the appropriate protein. (**c**) Negative-stain EM images of influenza A-B M2e-presenting SApNPs after heat treatment at 50, 60, and 70 °C (**d**) ELISA showing influenza A-B M2e-based immunogen binding to mAb148 and mAb65.

Figure S2

a **Mouse serum ELISA EC_{50} titers**

Antigen	EC50 titers (week 2)										Geometric Mean
	M1	M2	M3	M4	M5	M6	M7	M8	M9	M10	
M2exA3B3 1TD0 trimer	326.5	270.7	612.4	363.5	871	289.2	89.47	16.2	69.89	242.9	203.5
M2exA3B3 I3-01v9a SApNPs	2460	1535	1769	2592	3088	3459	1516	3932	2103	1771	2297.9
M2exB3A3 I3-01v9a SApNPs	2458	4069	2739	3447	4099	6917	2441	4928	2460	2112	3329.3
M2e(AB)x3 I3-01v9a SApNPs	1076	473.6	1660	1356	1792	2069	1252	1925	1044	1816	1345.4
M2e(BA)x3 I3-01v9a SApNPs	1484	968	1865	1444	1581	1410	4345	2430	1539	1219	1672.1

Antigen	EC50 titers (week 5)										Geometric Mean
	M1	M2	M3	M4	M5	M6	M7	M8	M9	M10	
M2exA3B3 1TD0 trimer	174152	13817	74732	20320	22758	8525	16548	5408	7568	8970	18340.7
M2exA3B3 I3-01v9a SApNPs	210684	180258	169830	199148	245451	203008	222892	193213	209258	83127	185385.1
M2exB3A3 I3-01v9a SApNPs	109611	83460	179999	222131	120769	102311	151243	177892	223975	117397	141409.2
M2e(AB)x3 I3-01v9a SApNPs	44747	90241	67911	122343	94670	130642	57744	110003	160877	114523	93031.1
M2e(BA)x3 I3-01v9a SApNPs	84970	42554	70252	79832	80588	113303	121094	84294	100677	74586	82263.3

b **Statistical analysis**

Week 2

One-way ANOVA with Tukey's multiple comparisons test (w2)	Statistics	Adjusted P Value
M2exA3B3 1TD0 trimer-w2 vs. M2exA3B3 I3-01v9a SApNPs-w2	****	<0.0001
M2exA3B3 1TD0 trimer-w2 vs. M2exB3A3 I3-01v9a SApNPs-w2	****	<0.0001
M2exA3B3 1TD0 trimer-w2 vs. M2e(AB)x3 I3-01v9a SApNPs-w2	ns	0.0604
M2exA3B3 1TD0 trimer-w2 vs. M2e(BA)x3 I3-01v9a SApNPs-w2	**	0.0051
M2exA3B3 I3-01v9a SApNPs-w2 vs. M2exB3A3 I3-01v9a SApNPs-w2	ns	0.056
M2exA3B3 I3-01v9a SApNPs-w2 vs. M2e(AB)x3 I3-01v9a SApNPs-w2	ns	0.1387
M2exA3B3 I3-01v9a SApNPs-w2 vs. M2e(BA)x3 I3-01v9a SApNPs-w2	ns	0.5993
M2exB3A3 I3-01v9a SApNPs-w2 vs. M2e(AB)x3 I3-01v9a SApNPs-w2	****	<0.0001
M2exB3A3 I3-01v9a SApNPs-w2 vs. M2e(BA)x3 I3-01v9a SApNPs-w2	***	0.001
M2e(AB)x3 I3-01v9a SApNPs-w2 vs. M2e(BA)x3 I3-01v9a SApNPs-w2	ns	0.8826

Week 5

One-way ANOVA with Tukey's multiple comparisons test (w5)	Statistics	Adjusted P Value
M2exA3B3 1TD0 trimer-w5 vs. M2exA3B3 I3-01v9a SApNPs-w5	****	<0.0001
M2exA3B3 1TD0 trimer-w5 vs. M2exB3A3 I3-01v9a SApNPs-w5	****	<0.0001
M2exA3B3 1TD0 trimer-w5 vs. M2e(AB)x3 I3-01v9a SApNPs-w5	*	0.0124
M2exA3B3 1TD0 trimer-w5 vs. M2e(BA)x3 I3-01v9a SApNPs-w5	ns	0.0808
M2exA3B3 I3-01v9a SApNPs-w5 vs. M2exB3A3 I3-01v9a SApNPs-w5	ns	0.1773
M2exA3B3 I3-01v9a SApNPs-w5 vs. M2e(AB)x3 I3-01v9a SApNPs-w5	***	0.0001
M2exA3B3 I3-01v9a SApNPs-w5 vs. M2e(BA)x3 I3-01v9a SApNPs-w5	****	<0.0001
M2exB3A3 I3-01v9a SApNPs-w5 vs. M2e(AB)x3 I3-01v9a SApNPs-w5	ns	0.0849
M2exB3A3 I3-01v9a SApNPs-w5 vs. M2e(BA)x3 I3-01v9a SApNPs-w5	*	0.0132
M2e(AB)x3 I3-01v9a SApNPs-w5 vs. M2e(BA)x3 I3-01v9a SApNPs-w5	ns	0.944

Fig. S2. Immunogenicity of influenza A-B M2e-based vaccines in mice. (**a**) EC_{50} titers for M2exA3B3 trimer and I3-01v9a SApNP vaccine-immune sera binding to sequence-matched M2e coating antigen (M2exA3B3-5GS-foldon, M2exB3A3-5GS-foldon, M2e(AB)x3-5GS-foldon, and M2e(BA)x3-5GS-foldon). Color coding indicates the magnitude of EC_{50} titers (green to red: low to high). (**b**) Table of statistical analysis was performed using a one-way ANOVA followed by Tukey's multiple-comparison *post hoc* test for each timepoint. EC_{50} titers were calculated in GraphPad Prism 10.2.3. For significance, ns (not significant), $*p < 0.05$, $**p < 0.01$, $***p < 0.001$, and $****p < 0.0001$.
